# Machine-learning based investigation of prognostic indicators for oncological outcome of pancreatic ductal adenocarcinoma

**DOI:** 10.3389/fonc.2022.895515

**Published:** 2022-12-08

**Authors:** Jeremy Chang, Yanan Liu, Stephanie A. Saey, Kevin C. Chang, Hannah R. Shrader, Kelsey L. Steckly, Maheen Rajput, Milan Sonka, Carlos H. F. Chan

**Affiliations:** ^1^ Department of Surgery, University of Iowa Hospitals and Clinics, Iowa City, IA, United States; ^2^ Iowa Initiative for Artificial Intelligence, University of Iowa, Iowa City, IA, United States; ^3^ Holden Comprehensive Cancer Center, University of Iowa, Iowa City, IA, United States; ^4^ Department of Radiology, University of Iowa Hospitals and Clinics, Iowa City, IA, United States; ^5^ Department of Electrical and Computer Engineering, University of Iowa, Iowa City, IA, United States

**Keywords:** machine learning, neural network, pancreatectomy, pancreatic cancer, surgical outcome, radiomics

## Abstract

**Introduction:**

Pancreatic ductal adenocarcinoma (PDAC) is an aggressive malignancy with a poor prognosis. Surgical resection remains the only potential curative treatment option for early-stage resectable PDAC. Patients with locally advanced or micrometastatic disease should ideally undergo neoadjuvant therapy prior to surgical resection for an optimal treatment outcome. Computerized tomography (CT) scan is the most common imaging modality obtained prior to surgery. However, the ability of CT scans to assess the nodal status and resectability remains suboptimal and depends heavily on physician experience. Improved preoperative radiographic tumor staging with the prediction of postoperative margin and the lymph node status could have important implications in treatment sequencing. This paper proposes a novel machine learning predictive model, utilizing a three-dimensional convoluted neural network (3D-CNN), to reliably predict the presence of lymph node metastasis and the postoperative positive margin status based on preoperative CT scans.

**Methods:**

A total of 881 CT scans were obtained from 110 patients with PDAC. Patients and images were separated into training and validation groups for both lymph node and margin prediction studies. Per-scan analysis and per-patient analysis (utilizing majority voting method) were performed.

**Results:**

For a lymph node prediction 3D-CNN model, accuracy was 90% for per-patient analysis and 75% for per-scan analysis. For a postoperative margin prediction 3D-CNN model, accuracy was 81% for per-patient analysis and 76% for per-scan analysis.

**Discussion:**

This paper provides a proof of concept that utilizing radiomics and the 3D-CNN deep learning framework may be used preoperatively to improve the prediction of positive resection margins as well as the presence of lymph node metastatic disease. Further investigations should be performed with larger cohorts to increase the generalizability of this model; however, there is a great promise in the use of convoluted neural networks to assist clinicians with treatment selection for patients with PDAC.

## Introduction

Pancreatic cancer is currently the third leading cause of cancer-related death in Western societies with an average annual incidence rate of 12.9 cases per 100,000 but a disproportionately high mortality rate of 10.9 deaths per 100,000 (1). Pancreatic ductal adenocarcinoma (PDAC) is the most common type of pancreatic cancer. At the time of diagnosis, only ~10% of PDAC are localized since small early cancers are often asymptomatic and left undiagnosed (2). Although surgery is the only curative treatment for PDAC, only 15%–20% of patients are candidates for surgical resection due to late presentation (2). The decision for upfront surgical resection followed by adjuvant chemotherapy vs. neoadjuvant treatment followed by surgical resection is based on both the anatomy of the tumor (i.e., vascular involvement) and risk stratification/prognostic features including the health condition, blood tumor markers, and lymph node involvement on imaging studies (3). Currently, computerized tomography (CT) scan is the most utilized modality for the evaluation of PDAC with a specificity and sensitivity of ~89% and ~90% (2). The nodal status is a well-established prognostic indicator for both overall survival and disease recurrence (4–6). Although CT image resolution has increased dramatically over the last two decades, the ability of a CT scan to assess both vascular invasion (sensitivity and specificity of 60% and 94%) and the nodal status remains suboptimal (positive predictive value and negative predictive value are 68% and 43.1%, respectively), as it may heavily depend on physician experience (1, 7). An automated prediction model for the presence of lymph node metastatic disease may preoperatively aid in clinical decision-making.

The choice to undergo the upfront surgical treatment of PDAC is determined by the preoperative CT stratification of resectability that is dependent on tumor proximity to the surrounding vessels (portal vein, superior mesenteric vein, superior mesenteric artery, and celiac artery) (8). The impact of the R1 resection status (i.e., presence of microscopic disease), defined as the distance of a tumor from the resection margin of less than or equal to 1 mm, on overall survival and recurrence-free survival is controversial (9–11). However, recent studies have suggested that the presence of microscopic disease within 1 mm is associated with decreased overall survival and decreased disease-free survival in PDAC in comparison to R0 resection (i.e., free of cancer cells at the resection margin) (12). Hong et al. demonstrated that of patients with the designation of a “resectable” tumor based on preoperative CT imaging, only 73% of patients had postoperative R0 resection on pathology (13). Preoperative CT appeared to overpredict resectability in tumors with any level of portomesenteric vein abutment and for larger tumors greater than 4 cm (12). An enhanced preoperative prediction of the surgical margin status would allow for improved patient selection for upfront curative intent surgery and importantly direct patients with tumors more likely to have postoperative R1 or R2 resection to neoadjuvant chemotherapy.

Radiomics is a novel approach to medical imaging that abstracts vast amounts of qualitative imaging features using data-characterizing algorithms, converting medical images into big data (14). The basis of the application of radiomics is that distinct imaging features between disease forms may be used to predict a prognosis and a therapeutic response (15). With radiomics exponentially increasing the data obtained from medical imaging, there has been growing interest with utilizing artificial intelligence or machines learning models to provide techniques to analyze these image data (16). One such model demonstrating great utility is the convoluted neural network (CNN). CNNs contain multiple interconnected layers of artificial neurons whereby each neuron can take an input, perform a computation, and produce output, while learning increases its higher-level functions (17). CNNs have been utilized to investigate a number of medical imaging questions including segmentation (i.e., tumor vs. normal tissue (18)), disease classification (19), detection and localization (i.e., identification of cerebral microbleeds in MRI (20)), and registration (i.e., integrating multiple scans of same patient (21)). Some examples include the following: Huang et al. have described that specific radiomic signatures differed between normal lymph nodes and lymph nodes with metastatic disease and that these differences allowed the creation of a nomogram for the prediction of the lymph node status in colorectal cancer (22). Chen et al. created hybrid many-objective radiomics and a three-dimensional CNN (3D-CNN) model to evaluate lymph node metastasis in head and neck cancers (23).

This paper proposes a novel machine learning predictive model, utilizing a 3D-CNN, to reliably predict the presence of lymph node metastasis and the postoperative positive margin status based on preoperative CT scans. This is the first deep learning predictive model for both lymph node disease in pancreatic cancer and the margin status based on preoperative imaging. Manual image segmentation was not performed allowing for an unbiased approach and a potential generalizability of the model to other abdominal/gastrointestinal cancers.

## Materials and methods

### Study population

The Biospecimen Procurement and Molecular Epidemiology Core (BioMER) is a shared core resource at the University of Iowa Holden Comprehensive Cancer Center that prospectively enrolls cancer patients into disease-specific MER patient cohorts annotated with clinicopathological, treatment, and outcome data. Within the gastrointestinal cancer cohort of the BioMER (GIMER), 462 patients were enrolled from 2015 to 2021. Study inclusion criteria included 1) having a diagnosis of pancreatic ductal adenocarcinoma by pathology, 2) receiving curative intent surgery, 3) available CT images prior to surgical intervention, and 4) available surgical pathologic data regarding the tumor margin status and lymph nodes. Positive margin was defined by the presence of cancer cells found within 1 mm from the inked resection margin. CT images and clinical and pathologic data were obtained from 110 patients (**Table 1**). A total of 881 CT scans were obtained. A patient’s CT scan from a particular date may contain the images of arterial, venous, and delayed phases with different resolutions. For the purposes of subgrouping, the images from each individual phase are classified as “one” scan. Due to small patient numbers, each scan was treated independently. The patient cohort was divided into two groups, one for training and one for validation for each study algorithm, margin study, and lymph node study. The training vs. validation split was 59 patients (340 scans) vs. 20 patients (140 scans) for the lymph node study and 83 patients (629 scans) vs. 27 patients (252 scans) for the margin study. For the margin study, additional PDAC patients with surgeon-determined unresectable locally advanced disease on preoperative CT were included to provide additional control cases with positive margin to improve study power.

**Table 1:** Study population characteristics.

	Lymph node study		Margin study	
	Training group (n=59)	Validation group (n=20)	Training group (n=83)*	Validation group (n=27)^§^
**Age**	66.1 [63.6–68.7]	62.5 [58.6–66.3]	65.8 [63.6–68.0]	64.2 [60.5–67.8]
**Gender**
Male	27 (45.8%)	10 (50%)	44 (53%)	13 (48.1%)
Female	32 (54.2%)	10 (50%)	39 (47%)	14 (51.9%)
**Pathological Stage**
Stage 0	0	1 (5%)	0	1 (3.7%)
Stage I	5 (8.5%)	2 (10%)	5 (6.0%)	2 (7.4%)
Stage II	54 (91.5%)	17 (85%)	55 (66.3%)	16 (59.3%)
Stage III	0	0	16 (19.3%)	6 (22.2%)
Stage IV	0	0	7 (8.4%)	2 (7.4%)
**Positive Margin**	12 (20.3%)	4 (20%)	36 (44.4%)	11 (40.7%)
**Number of Images**	340	140	629	252

*Includes 23 unresectable cases (these cases would yield a positive resection margin if they have undergone surgery).

^§^Includes 8 unresectable cases.

### Development of machine learning algorithm

In collaboration with the Iowa Initiative for Artificial intelligence (IIAI), a 3D-CNN was developed for the purpose of image classification based on the lymph node disease or the margin status. In a basic sense, the CNN involves creating a scaffolding of computational “layers” stacked on one another whereby the outputs of terminal layers are built upon the inputs of the previous. The specific structuring of the number of layers and the type of layer (i.e., convolutional, pooling, and fully connected) based on the research question is where nuance arises. The goal was to learn a discriminative function, f ∈ {0, 1}, where 1 indicates lymph node metastasis or a positive margin and 0 otherwise.

**Figure 1:**
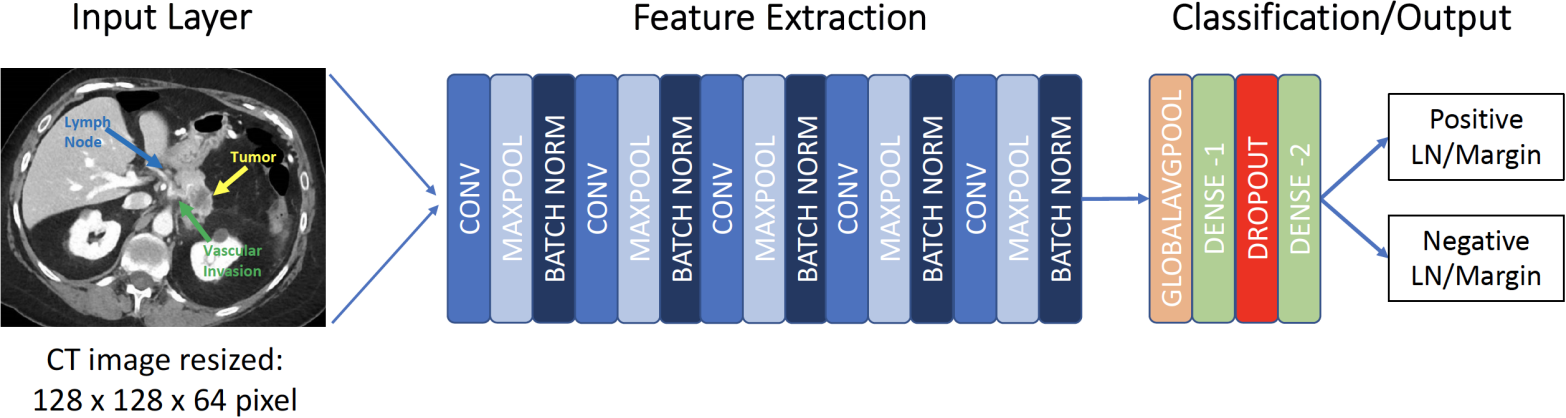
Framework for 3D convolutional neural network. CONV, convolutional layer; MAXPOOL, max pooling; LN, Lymph node.

The 3D-CNN utilized was modeled after that described by Zunair et al. (24). Like Zunair et al, this study utilized a 17-layer 3D-CNN. Four 3D convolutional layers are used with each convolutional layer followed by a max-pooling layer and a subsequent batch normalization layer creating a CON-MAXPOOL-BN module (24). The subsequent output runs through a global average pooling layer and then a dense layer. An effective dropout rate of 60% was utilized. A second dense layer was used to produce output consistent with the binary classification problem (**Figure 1**). The binary cross-entropy loss function was utilized within model learning to optimize the performance of the classification model. A total of 1,351,813 learnable parameters were present in this study. All codes were written and run utilizing Python (Python Software Foundation, Delaware, USA).

## Data preparation

To decrease computational time, slice selection was performed. Each axial CT scan was analyzed, and the slices between an anatomical boundary of superior to the celiac artery takeoff to inferior to the renal vein were identified. Subsequently, each image was resized to a resolution of 128 × 128 × 64 pixels. Image intensity and parameters were normalized to a scale of (0, 1). The initial input for the first layer of the 3D-CNN model was resized CT scan.

### Statistical analysis

The sensitivity, specificity, positive and negative predictive values, and accuracy of the model were evaluated on training and validation datasets. With the use of the Wilson–Brown Method with GraphPad Prism8 software, 95% confidence intervals (17) were determined. Receiver operating characteristic (25) curves were plotted for the per-patient analysis using the different cutoff values of percent-positive scan from per-scan analysis for each patient, and area-under-the-curve (26) analysis was performed using GraphPad Prism8 software. Algorithm prediction accuracy was displayed in the confusion matrix as appropriate.

## Results

The clinical characteristics of study population are summarized in **Table 1**.

### Lymph node metastasis predictive model

**Figure 2:**
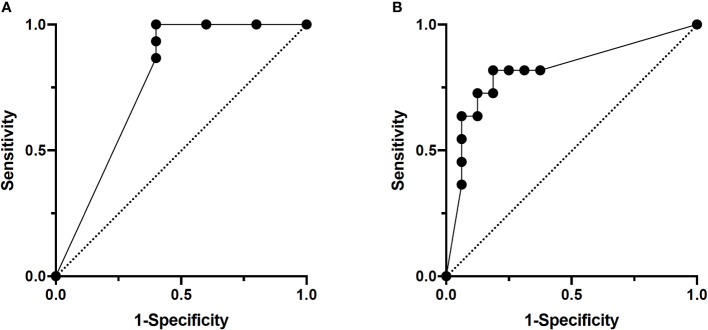
ROC curves of per-patient analysis. **(A)** Lymph node study. AUC: 0.79. **(B)** Margin study. AUC: 0.85.

The training group consisted of 37 patients with lymph node metastasis and 22 patients without (total of 340 scans), and the validation group consisted of 15 patients with lymph node metastasis and 5 patients without (total of 140 scans). In per-scan analysis, the 3D-CNN model achieved a sensitivity of 93% (95%CI: 86%–97%) and a specificity of 42% (95%CI: 29%–56%) with an accuracy of prediction at 75% and a positive and negative predictive value of 74% (95%CI: 66%–82%) and 78% (95%CI: 59%–89%), respectively (**Table 2**). Using majority voting strategy in per-patient analysis, the 3D-CNN model achieved a sensitivity of 100% (95%CI: 80%–100%) with a specificity of 60% (95%CI: 23%–93%) with an accuracy of 90% and a positive and negative value of 88% (95%CI: 66%–98%) and 100% (95%CI: 44%–100%), respectively (**Table 2**). Using various cutoff values in per-patient analysis, an ROC curve was constructed with an AUC of 0.786 (95%CI: 0.510–1.000) (**Figure 2A**) and the best cutoff value was indeed the same as the major voting strategy (i.e., >50% of scans predicted to be positive).

**Table 2:** Confusion matrix for lymph node study.

Type of analysis	True positive	True negative
**Per-patient analysis (n=20)**		
Predicted Positive	15	2
Predicted Negative	0	3
**Per-scan analysis (n=140)**		
Predicted Positive	84	29
Predicted Negative	6	21

### Postoperative positive-margin predictive model

The training group consisted of 83 patients (total of 629 scans) with 36 patients having a positive margin. The validation group for the margin model consisted of 27 patients (total of 252 scans), 11 of whom had a positive margin. In per-scan analysis, the 3D-CNN model achieved a sensitivity of 67% (95%CI: 59%–74%) and a specificity of 89% (95%CI: 81%–93%) with an accuracy of 76% and a positive and negative predictive value of 89% (95%CI: 82%–94%) and 65% (95%CI: 57%–73%), respectively (**Table 3**). Using majority voting strategy in per-patient analysis, the 3D-CNN model achieved a sensitivity of 73% (95%CI: 43%–90%) and a specificity of 88% (95%CI: 64%–98%) with accuracy of 81% and a positive and negative predictive value of 80% (95%CI: 49%–96%) and 82% (95%CI: 59%–94%), respectively (**Table 3**). Using various cutoff values in per-patient analysis, an ROC curve was constructed with an AUC of 0.852 (95%CI: 0.670–1.000) (**Figure 2B**) and the best cutoff values were between 40% and 60%.

**Table 3:** Confusion matrix for margin study.

Type of analysis	True positive	True negative
**Per-patient analysis (n=27)**		
Predicted Positive	8	2
Predicted Negative	3	14
**Per-scan analysis (n=252)**		
Predicted Positive	98	12
Predicted Negative	49	93

## Discussion

The purpose of this study is to provide a proof of concept that 3D CNN-based algorithms can predict lymph node metastasis and the postoperative margin status with clinically relevant levels of accuracy. The CT scans of 110 patients from a single tertiary care institution were utilized without segmentation. The lymph node prediction model achieved an accuracy of 75% in per-scan analysis and 90% in per-patient analysis using majority voting, while the postoperative margin prediction model achieved an accuracy of 76% in per-scan analysis and 81% in per-patient analysis using majority voting. This is the first study to utilize a 3D-CNN for the prediction of postoperative margins and the first study to utilize a 3D-CNN to predict the lymph node status in pancreatic cancer.

The most promising type of machine learning model for radiomic analysis has been the CNN (16). CNNs were developed in the late 1970s and saw their first application into medical imaging analysis in the 1990s (27). CNNs became more widely recognized after the ImageNet Large Scale Visual Recognition Challenge (ILSVRC) in 2012, whereby algorithms were tasked with classifying over 1.2 million high-resolution images from 22,000 categories into 1,000 classes (28). AlexNet, the winning model, was highly efficient and accurate and provided a framework for the future iterations of CNNs (29). CNNs are a more popular option in comparison to other types of machine learning algorithms, such as the random forest model or decision trees for radiomic data. They are superior in modeling non-linear relationships in seemingly unrelated data to achieve a result (30). In contrast to random forest models, CNNs lack the interpretability of individual features and focus on solving a specific problem (16, 31). CNNs have been applied to a wide range of medical problems with over 300 papers published in the last few years (17). All kinds of medical imaging including X-ray, CT, MRI, and ultrasound have been utilized with CNNs (17). For example, in pancreas imaging, studies have been performed looking to use 3D-CNNs for the diagnosis of pancreatic cystic neoplasms, neuroendocrine tumors, and additional segmentation of the pancreas. Recently in 2020, a 3D-CNN model was described for the classification of pancreatic cancer from initial diagnostic CT scans that demonstrated a sensitivity of 99% and an accuracy of 99%. Another study used CNNs to measure pancreas volumes in patients with type 1 diabetes (32). The pancreas is an inherently more difficult organ to evaluate than the liver or kidney due to its variable shape, size, and proximity to numerous structures. Studies utilizing pre-analysis segmentation to isolate the pancreas from neighboring structures have yielded improved accuracy in comparison to non-segmentation studies (33).

Lymph node metastasis is a significant prognostic factor in pancreatic cancer survival; however, preoperative lymph node identification remains a challenge in the diagnostic radiology of pancreatic and other abdominal cancers with sensitivities ranging from 40% to 87% and specificities ranging from 64% to 100% for CT and MRI (26, 34). Radiologists are limited to looking at the size, shape, and contour of lymph nodes on CT scans. Specifically, CT and MRI techniques are limited in the ability to detect metastatic disease in normal-sized or minimally enlarged lymph nodes. Based on tumor morphology, the incidence of metastatic disease within normal-sized nodes may occur anywhere from 10% to 90% of cases (35). In pancreatic cancer, the size of ≥1cm was only 44.2% sensitive to the identification of lymph node metastasis (34). While there have been attempts to utilize CNNs in CT segmentation to identify metastatic lymph nodes, the first use of CNNs to evaluate for potentially metastatic lymph nodes was performed in head and neck cancers by Chen et al. (36). Utilizing many-objective radiomics in conjunction with the 3D-CNN framework, the group created a model to predict three classes of lymph nodes: normal, suspicious, or involved. Segmentation was used to identify specific nodes for analysis. The accuracy of the model was 0.88. Additional machine learning models have been created for the identification of lymph node metastasis in cervical cancer (37) and the prediction of lymph node metastasis in gastric cancer (38) and prostate cancer (39). This is the first study investigating the pancreas. The 3D-CNN proposed by this paper offers a different approach as this model does not utilize segmentation and imaging studies were at a different anatomical location likely involving different radiomic parameters. An acceptable accuracy of 90% was achieved in per-patient analysis.

The accuracy of CT imaging for predicting resectability is approximately 70% and is prone to overestimation (40). The ability to improve preoperative patient selection for such a substantial surgical procedure could be vital in improving overall clinical outcomes. Patients who are deemed to be high risk for R1 resection even though their tumors are classified as technically resectable based on current clinical and radiological guidelines may warrant a consideration for neoadjuvant therapy. It remains controversial whether the postoperative positive microscopic margin (R1 resection) has an impact on survival postoperatively since the probability of the recurrence-free survival and overall survival of these patients depends on multiple factors including underlying medical conditions, the postoperative course, the choice of systemic treatment, the treatment response, pathological and molecular subtypes, and the stage of disease. The rates of R1 resection in the literature may range widely from as low as 16% to >75% with some studies also noting an association with poorer clinical outcomes in comparison to R0 resection but not others (41). This discrepancy was due to a lack of standardization with the pathologic evaluation of resection specimens and definitions (9). The Royal College of Pathologists define R1 resection as a “microscopic evidence of tumor within 1 mm of a resection margin” (42, 43). The adoption of the standardized definitions of R1 resection as well as the circumferential resection margin has led to increase in the literature-reported incidence rates of R1 resection (44). Recent meta-analysis data have shown that R1 resection is associated with decreased overall survival and disease-free survival in PDAC patients after pancreaticoduodenectomy (Whipple procedure) (9, 12). One thinking is that R1 resection following curative intent surgery may indicate the presence of micrometastatic disease unable to be identified preoperatively. A developing paradigm shift in the management of PDAC is the usage of neoadjuvant therapy in the cases of resectable or borderline resectable cancer (45). The Preoperative radiochemotherapy versus immediate surgery for resectable and borderline resectable pancreatic cancer (PREOPANC-1) randomized phase III trial, comparing neoadjuvant with gemcitabine and chemoradiation vs. adjuvant gemcitabine in resectable or borderline resectable tumors did not identify any difference in overall survival; however, there was improvement in the secondary endpoints of disease-free survival and the R0 resection rate (45). In subgroup analysis, borderline resectable but not resectable tumors demonstrated an improvement in overall survival (46). In this study population, survival analysis supports the notion that a positive resection margin is associated with worse overall survival and recurrence-free survival, as well as worse local and distant recurrence-free survival in Kaplan–Meier and univariate Cox hazard ratio analyses (**Supplemental Figure 1** and **Supplemental Tables 1**, **2**). The lymph node status was only associated with overall survival (**Supplemental Figure 2** and **Supplemental Tables 1**, **2**). In multivariate Cox hazard ratio analysis, a positive margin remains associated with recurrence-free survival (**Supplemental Table 2**). This suggests that the margin status may act as a surrogate marker of recurrence. It is important to note that in this study population, only 16 out of 110 patients received neoadjuvant therapy, with the majority of borderline resectable tumors receiving upfront surgery. The purpose of performing preoperative margin prediction is to potentially assist in clinical decision-making for these types of tumors, where patients predicted to have positive margin should probably consider neoadjuvant treatment instead of upfront surgery.

Margin studies are difficult to accomplish specifically in the pancreas due to the need for the CNN to understand and evaluate proximity to a “weighted” group of vital structures. There have been no machine-learning models trained to identify the postoperative margin status from preoperative images. A study performed by Halicek et al. described the use of CNNs in patients with squamous cell carcinoma in their oral cavity to identify residual disease on postresection imaging studies (47). The model proposed in this paper utilized a simplistic approach to provide a proof of concept with subsequent fine-tuning available in future iterations. Without the segmentation of images, the model learns the pancreas, auto-segments the tumor from the normal pancreas, and attempts to classify the characteristics of surrounding pixels to trained binary outcomes. A future iteration of the model should look to identify specific radiomic parameters investigated in order to compare the radiomic differences between high- and low-risk tumors for a postoperative positive margin.

A major limitation of this study is the small sample size for respective training and validation groups. In our algorithm, this was attempted to be mediated by additional per-scan analysis to increase sample size as well as well as limiting the number of trainable parameters, which demonstrated worse accuracy in comparison to majority voting in per-patient analysis. The concern when utilizing smaller datasets is whether the model is specifically learning features that would ideally distinguish from the testing criteria or just overfitting for some features in the given dataset. Additionally, the design of this study is such that the outcome is a binary yes or no to the question posed. There is no distinction to which the lymph node station or margin is the one predicted to be positive nor if the features of the true-positive lymph node or margin are sampled. A consideration for future modification to this 3D-CNN model would be to use postoperative lymph node pathology with preoperative image segmentation for individual lymph node stations and tumor boundaries in the training group. Additionally, future CNN models on larger datasets should seek to perform iterations with the optimization of overall survival and recurrence-free survival with propensity- matched cases to alleviate confounding characteristics. Lastly, a small dataset of 110 patients, majority (98%) Caucasian, could mean that training CT images may not be representative of the generalizable population of PDAC tumors (48). Additional diversity should be included in additional training groups for CNNs.

Medical outcome modeling for treatment planning is a novel application of convolutional neural networks that warrants additional investigation.

## Conclusion

In conclusion, this study provides a proof of concept that utilizing radiomics, the 3D-CNN deep learning framework may be used to improve the preoperative prediction of positive resection margins as well as the presence of lymph node metastatic disease. Further investigations should be performed with larger cohorts to increase the generalizability of this model; however, there is great promise in the use of CNNs to assist clinicians with treatment selection for patients with PDAC.

## Data availability statement

The raw data supporting the conclusions of this article will be made available by the authors, without undue reservation.

## Ethics statement

The studies involving human participants were reviewed and approved by Institutional Review Board, University of Iowa. Written informed consent for participation was not required for this study in accordance with the national legislation and the institutional requirements.

## Author contributions

JC performed clinical data abstraction, defined the boundaries of all CT scans, performed data analysis, and wrote the manuscript. YL prepared the image files, built the 3D-CNN models, and performed data analysis. SS and KC performed clinical data abstraction. HS and KS performed the abstraction of clinical and imaging data. MR offered radiology insights and provided supervision/guidance on defining boundaries on CT scans. MS offered insight and guidance on imaging analysis. CC conceptualized the overall study, supervised the study, provided clinical and surgical insights, obtained research funding, performed data analysis, interpreted experimental data, and edited the manuscript. All authors contributed to the manuscript and approved for the manuscript submission.
