# Supplementary material for: Machine-learning based investigation of prognostic indicators for oncological outcome of pancreatic ductal adenocarcinoma
Source: Front Oncol. 2022 Dec 8;12:895515. doi: 10.3389/fonc.2022.895515 (PMC9773248; doi:10.3389/fonc.2022.895515)
Supplement: Supplementary file 1 [file DataSheet_1.pdf]

Supplementary Material

1 Supplementary Data

Supplemental Methods

Kaplan-Meier analysis and univariate and multivariate Cox proportional hazard ratio analysis was performed to investigate associations between node and margin status with overall survival (OS), recurrence free survival (RFS), local recurrence free survival (LRFS) and distant recurrence free survival (DRFS).

Supplemental Results

Impact of Margin and Lymph Node Status on Overall Survival (OS) and Recurrence-Free Survival (RFS)

Survival analysis was performed on study population who received surgery. Patients were excluded if postoperative mortality was secondary to acute postoperative complication. In total, survival analysis was performed on 74 patients, of whom, 15 were noted to have positive resection margin and 49 had positive lymph nodes. 45 patient experienced disease recurrence after curative intent surgery, with 16 local recurrences and 37 distant recurrences (some patients had both). Median overall survival for patient with positive margin and positive lymph node was decreased compared to negative margin (14 months vs 27 months, p=0.015) and negative lymph node (22 months vs 29 months, p=0.044), respectively (Supplemental figure 1A and 2A). Positive margin was associated with decreased RFS (10 vs 19 months, p=0.0029) as well as local RFS (p=0.023) and distant RFS (p=0.0078), while there were no statistically significant differences between positive and negative lymph node status in Kaplan Meier survival analysis (Supplemental figure 1 and 2).

Univariate Cox proportional hazards model was utilized to investigate association between margin and lymph node status and overall survival, RFS, LRFS and DRFS (Supplemental Table 1). Both positive margin status (HR=2.10, p=0.016) and positive lymph node status (HR = 1.75, p=0.047) were associated with worse overall survival. Positive margin was associated with worse RFS (HR=2.68, p=0.004), LRFS (HR=3.28, p=0.030) and DRFS (HR=2.64, p=0.010), while lymph node status was not statistically significant. Multivariate analysis was performed adjusting for lymphovascular invasion, perineural invasion, stage, histological grade and gender (Supplemental Table 2). In multivariate analysis of OS, RFS, LRFS and DRFS, positive margin was only associated with RFS (HR=2.21, p-0.046).

2 Supplementary Figures and Tables

Supplemental Table 1: Univariate Cox Hazard Ratio Analysis

	Overall Survival		Recurrence Free Survival		Local Recurrence Free Survival		Distant Recurrence Free Survival	
	HR [95% CI]	*p-value*	*HR [95% CI]*	*p-value*	*HR [95% CI]*	*p-value*	*HR [95% CI]*	*p-value*
**Male Gender**	1.058 [0.645 - 1.740]	0.823	1.069 [0.591 - 1.933]	0.825	1.008 [0.375 - 2.710]	0.987	1.453 [0.740 - 2.856]	0.278
**Age > 65**	0.778 [0.474 - 1.278]	0.322	0.619 [0.342 - 1.118]	0.112	0.506 [0.184 - 1.394]	0.188	0.646 [0.337 - 1.238]	0.188
**Positive Margin**	2.103 [1.152 - 3.840]	**0.016**	2.689 [1.375 - 5.259]	**0.004**	3.283 [1.125 - 9.585]	**0.030**	2.643 [1.265 - 5.524]	**0.010**
**Positive Nodes**	1.746 [1.008 - 3.025]	**0.047**	1.750 [0.903 - 3.393]	0.098	1.874 [0.604 - 5.818]	0.277	1.980 [0.933 - 4.202]	0.075
**Lymphovascular Invasion**	1.550 [0.896 - 2.683]	0.117	2.005 [0.990 - 4.061]	0.054	1.616 [0.519 - 5.029]	0.407	2.439 [1.068 - 5.572]	**0.034**
**Perineural Invasion**	1.781 [0.926 - 3.426]	0.084	1.245 [0.600 - 2.587]	0.556	2.155 [0.490 - 9.487]	0.310	0.971 [0.458 - 2.058]	0.938
**Stage (relative to pT1)**								
**pT2**	0.957 [0.292 - 3.314]	0.942	1.639 [0.347 - 7.733]	0.533	0.430 [0.027 - 6.880]	0.551	1.619 [0.343 - 7.648]	0.543
**pT3**	1.360 [0.488 - 3.785]	0.557	1.474 [0.354 - 6.135]	0.594	0.850 [0.155 - 8.950]	0.875	1.124 [0.267 - 4.733]	0.874
**Neoadjuvant Therapy**	0.917 [0.498 - 1.688]	0.780	1.263 [0.6395 - 2.494]	0.501	1.7345 [0.602 - 4.995]	0.308	1.254 [0.591 - 2.658]	0.556
**Chemotherapy**	1.373 [0.778 - 3.196]	0.462	1.282 [0.506 - 3.251]	0.601	1.147 [0.261 - 5.053]	0.856	1.314 [0.465 - 3.711]	0.607
**Radiotherapy**	0.982 [0.598 - 1.612]	0.943	1.127 [0.630 - 2.026]	0.690	1.273 [0.473 - 3.423]	0.632	0.926 [0.486 - 1.767]	0.816

Supplemental Table 2: Multivariate Cox Hazard Ratio Analysis

	Overall Survival		Recurrence Free Survival		Local Recurrence Free Survival		Distant Recurrence Free Survival	
	HR	p-value	HR	p-value	HR	p-value	HR	p-value
**Positive Margin**	1.612	0.173	2.214	**0.046**	2.033	0.312	2.279	0.0658
**Positive Nodes**	0.486	0.62	1.841	0.997	5.215	0.988	3.523	0.998
**Lymphovascular Invasion**	1.478	0.185	1.444	0.300	1.342	0.654	2.032	0.110

Supplementary Figure 1

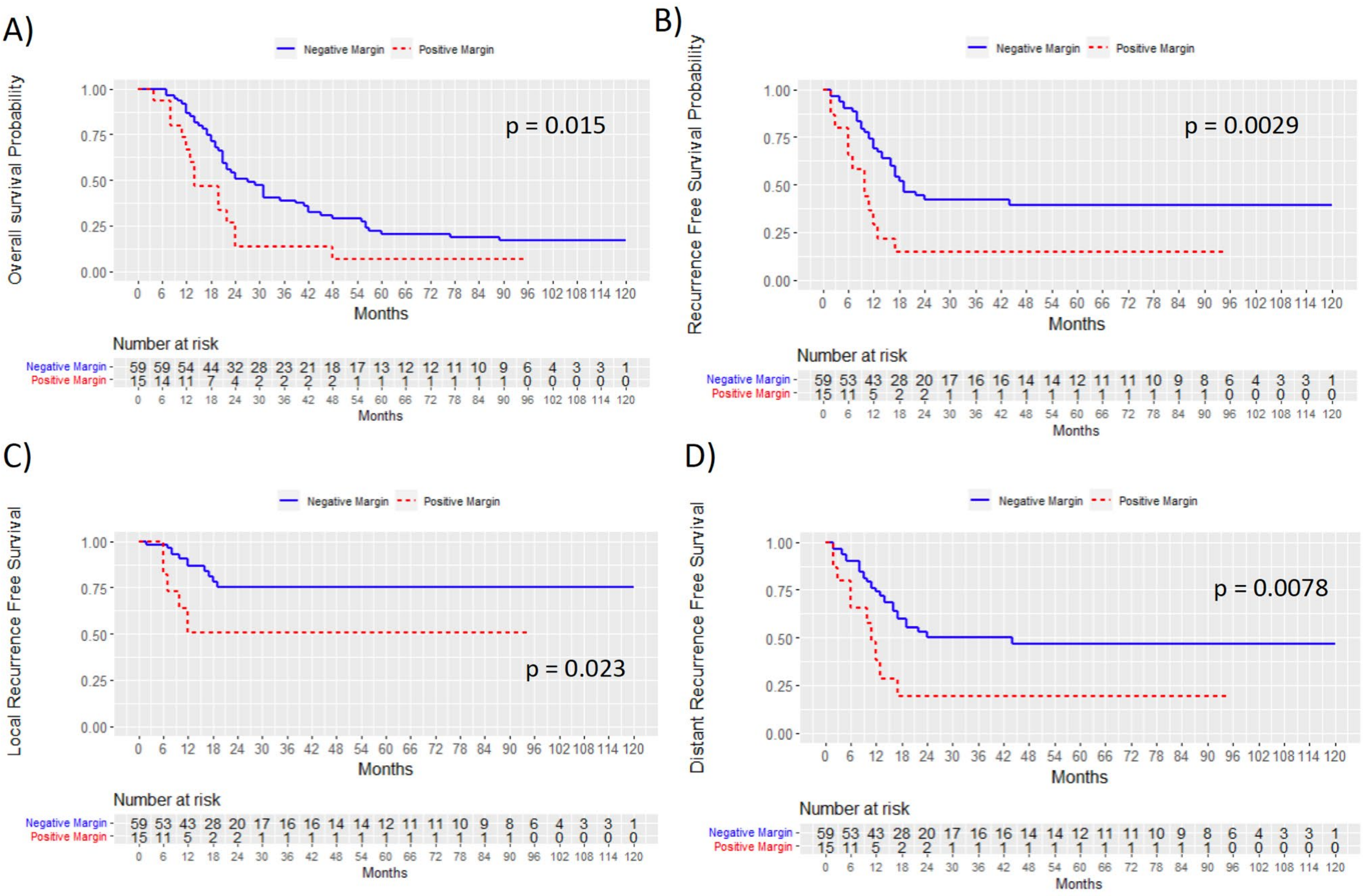

Supplemental Figure 2

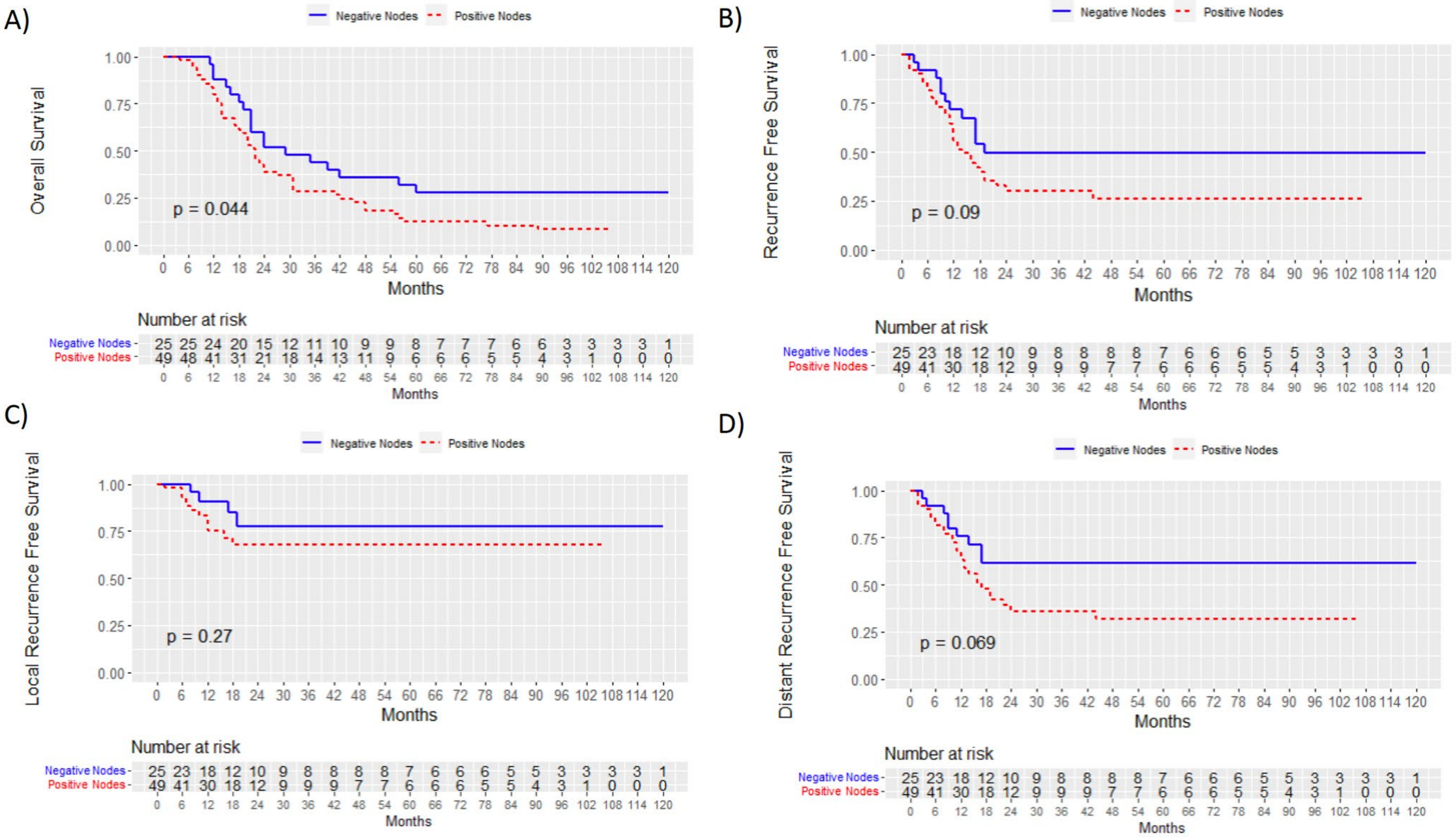

Supplemental Figure Legends

Supplemental figure 1: Kaplan Meier Survival Analysis of Margin Status for A) Overall Survival, **B)** Recurrence Free Survival, **C)** Local Recurrence Free Survival and **D)** Distant Recurrence Free Survival.

Supplemental figure 2: Kaplan Meier Survival Analysis of Lymph Node Status for A) Overall Survival, **B)** Recurrence Free Survival, **C)** Local Recurrence Free Survival, **D)** Distant Recurrence Free Survival.
